# High-resolution lensless holographic microscopy using a physics-aware deep network

**DOI:** 10.1117/1.JBO.29.10.106502

**Published:** 2024-10-08

**Authors:** Ashwini S. Galande, Vikas Thapa, Aswathy Vijay, Renu John

**Affiliations:** Indian Institute of Technology Hyderabad, Department of Biomedical Engineering, Medical Optics and Sensors Laboratory, Hyderabad, Telangana, India

**Keywords:** lensless holography, physics-aware neural networks, cervical cells, high-resolution

## Abstract

**Significance:**

Lensless digital inline holographic microscopy (LDIHM) is an emerging quantitative phase imaging modality that uses advanced computational methods for phase retrieval from the interference pattern. The existing end-to-end deep networks require a large training dataset with sufficient diversity to achieve high-fidelity hologram reconstruction. To mitigate this data requirement problem, physics-aware deep networks integrate the physics of holography in the loss function to reconstruct complex objects without needing prior training. However, the data fidelity term measures the data consistency with a single low-resolution hologram without any external regularization, which results in a low performance on complex biological data.

**Aim:**

We aim to mitigate the challenges with trained and physics-aware untrained deep networks separately and combine the benefits of both methods for high-resolution phase recovery from a single low-resolution hologram in LDIHM.

**Approach:**

We propose a hybrid deep framework (HDPhysNet) using a plug-and-play method that blends the benefits of trained and untrained deep models for phase recovery in LDIHM. The high-resolution phase is generated by a pre-trained high-definition generative adversarial network (HDGAN) from a single low-resolution hologram. The generated phase is then plugged into the loss function of a physics-aware untrained deep network to regulate the complex object reconstruction process.

**Results:**

Simulation results show that the SSIM of the proposed method is increased by 0.07 over the trained and 0.04 over the untrained deep networks. The average phase-SNR is elevated by 8.2 dB over trained deep models and 9.8 dB over untrained deep networks on the experimental biological cells (cervical cells and red blood cells).

**Conclusions:**

We showed improved performance of the HDPhysNet against the unknown perturbation in the imaging parameters such as the propagation distance, the wavelength of the illuminating source, and the imaging sample compared with the trained network (HDGAN). LDIHM, combined with HDPhysNet, is a portable and technology-driven microscopy best suited for point-of-care cytology applications.

## 1 Introduction

Quantitative phase imaging (QPI) has enabled label-free quantitative investigations of biological cells and tissues by measuring their optical, chemical, and mechanical properties.^1^ Digital holographic microscopy (DHM), a QPI modality, is an interferometric technique for measuring morphological characteristics along with dynamics.^2^^,^^3^ The core idea of DHM is that a coherent light wave, on passing through an object, experiences diffraction and change of phase induced by the object. This information is captured in the interference pattern formed by the superposition of the wave diffracted/scattered by the object and the un-scattered wave at some angle. The goal is to generate a 3D shape of the object by processing the captured hologram. However, the stringent requirements of a coherent source, environmental stability, high numerical aperture (NA) microscope objectives, and device complexity limit its use in low resource settings and point-of-care applications. The development of a low-cost, portable, and technology-driven imaging modality is highly desirable for point-of-care applications. The real-time analysis allows for regular clinical check-ups and early diagnosis of severe diseases such as cancer, anemia, and bacterial infections, which can save many lives in rural areas.

**Fig. 1:**
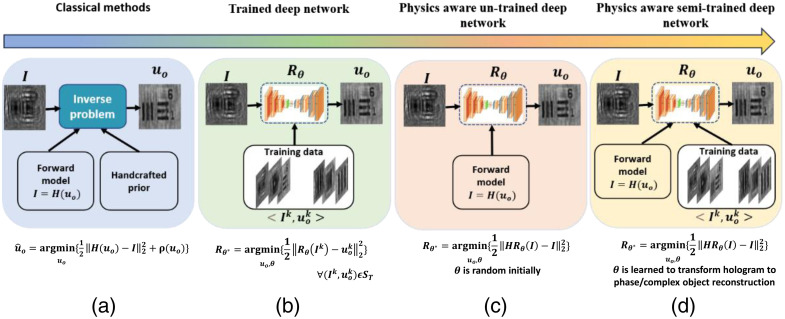
(a)–(d) Hologram reconstruction based on inverse problem approach: classic versus trained deep models versus un-trained deep models.

With the recent advances in computational capability, reconstruction algorithms, and faster imaging devices with low cost, the concept of lensless holographic microscopy^4^^,^^5^ has emerged as an attractive alternative to the traditional DHM.^6^ Using a partially coherent source in lensless digital inline holographic microscopy (LDIHM) enables portable, low-cost, and speckle-free imaging devices for point-of-care applications.^7^^,^^8^ However, twin image artifacts, which are caused by the propagation of the conjugate wavefront and the resolution limit, make the reconstruction challenging with single-shot hologram recording. Hologram reconstruction is formulated as an inverse problem (IP) approach that searches for the reconstructed object, in the solution space, that is most consistent with the captured hologram given some prior knowledge of the object under reconstruction (as shown in Fig. 1).^9^ This is an ill-posed problem due to the availability of multiple solutions and requires regularization $\left[\rho (u_{o})\right]$. Methods such as Hand-crafted priors,^9^ sparsity,^10^ and learned priors^11^^,^^12^ are imposed as a constraint along with the data fidelity term in the minimization process. Several optimization methods have been proposed to solve this optimization problem.^13^^–^^16^ However, the reconstruction quality is highly dependent on the choice of the suitable handcrafted prior, the optimal tuning parameter, and the heuristic search that may get stuck at local optima. Recent studies have shown interest in combining the alternating projections strategy (phase retrieval methods) with the inverse problem approach for single-shot phase reconstruction.^17^ However, convergence on a single low-resolution hologram with a large field-of-view (FOV) is challenging.

Deep learning (DL) methods can effectively solve inverse hologram reconstruction problems with superior performance over conventional methods.^18^^–^^21^ Based on the requirement of training data, we provide a taxonomy (as shown in Fig. 1) for the classification of DL methods in hologram reconstruction. In Fig. 1, H represents the forward imaging model, $u_{o}$ is the object under reconstruction, $R_{\theta }$ represents the deep network with θ as the learnable parameters, and I is the captured hologram. $\rho (u_{o})$ is the handcrafted regularization/constraint on the object under reconstruction. In the classic inverse problem approach [Fig. 1(a)], the reconstruction is formulated as the optimization problem in which the error between the predicted and captured hologram is minimized while following the constraint on the reconstructed object. However, the handcrafted priors often fail to capture the rich structure of many natural signals due to a lack of discriminative power.

Trained/supervised DL methods to solve inverse problems have demonstrated impressive performance in computational imaging.^22^ Here, the network $R_{\theta }$ learns the mapping between the input hologram $\left(I^{k}\right)$ and the reconstructed object $\left(u_{o}\right)$ from the training dataset $\left(S_{T}\right)$. Hologram reconstruction based on supervised DL has shown significant improvement in the reconstructed phase from a single hologram.^18^^,^^23^ Despite the superior performance of supervised DL methods in phase recovery, they suffer from the drawback of the requirement of a large, object-specific training data pair $\left(I^{k},u_{o}^{k}\right)$, which is very difficult to achieve. Also, these models learn the morphological characteristics of the samples for pixel-to-pixel transformation, which may lead to failure for the patterns that were not provided in the training dataset. These data-driven end-to-end networks do not consider the physics of the hologram formation process (imaging physics).^18^^–^^21^

To alleviate the data requirement problems, recently proposed untrained deep models incorporate the physics of hologram formation in the hologram reconstruction process.^24^^–^^30^ The network could learn to reconstruct 3D objects from holographic data by directly capturing patterns and relationships from the input. As shown in Fig. 1(c), the error between the captured and measured holograms is used to update the network parameters (θ). The interplay between the network ($R_{\theta }$) and the forward model (H(I)) learns the network parameters. These models aim to leverage the expressive power of deep networks while ensuring that the reconstructed object adheres to the fundamental laws of wave propagation and interference. However, the process of fitting reconstruction to the single measured hologram results in noisy reconstruction. Explicit regularization methods have been proposed to improve the reconstruction by eliminating noise.^30^^,^^31^ However, we observed in our previous work^31^ that the untrained networks perform better for low-complexity signals as they lack the learned knowledge and predictive capabilities that come with training. Hence, the synergy of supervised deep models with the physics of holography is highly desirable for the hologram reconstruction process (we refer to them as semi-trained models).

The recently proposed semi-trained methods can refine results and improve interpretability by leveraging the ability of deep networks to extract meaningful representations from the limited data and constraining the fundamental laws of wave propagation and interference on the reconstruction.^32^^,^^33^ Here, θ is learned from the limited dataset and further improved with the physics consistency. Based on the model architecture and type of dataset required for pre-training, we roughly categorized these methods into three groups: unpaired training approach, model-based deep unfolding approach, and plug-and-play (PnP) approach. The unpaired training approach uses the cycle consistency loss and a generative adversarial network (CycleGAN) in which the real distribution of the object is not necessary and hologram and ground truth are irrelevant. The CycleGAN-based reconstruction of holograms is robust against the aberrations present in imaging and can be used for real-time reconstruction.^34^^,^^35^ Model-based deep unfolding networks are designed to imitate the behavior of the conventional alternate projection optimization methods. Each stage consists of a trainable deep network that implements one iteration of the alternating projection method. Furthermore, the physics-based constraints enforced in each stage/iteration benefit from the representational capabilities of deep networks as well as the interpretability of traditional iterative algorithms.^36^ The third approach, the PnP method, makes use of the alternating directions of multipliers method (ADMM) to combine DL and denoiser of a choice into a robust single-shot phase recovery process.^37^^,^^38^ ADMM allows for alternating between a data fidelity term and the denoiser, which promotes parallelism to reduce the computational time.

In this paper, we address two different problems of LDIHM reconstruction: the twin-image free phase recovery from a single-shot hologram and improving the resolution, which is limited by the pixel size of the camera. We propose a hybrid approach using the PnP model that combines the phase recovered by the high-definition GANs (HDGANs)^39^ as *a priori* to the physics-aware deep networks (UNet).^31^ We refer to our proposed method as HDPhysNet. The flexibility of the PnP models, which use the ADMM for optimization, makes it possible to plug the learned regularization in the loss function along with the data fidelity term without explicit differentiation. The learned prior inherently denoises the twin image and upsamples the reconstructed phase image by 2× or 4× while leveraging the ability of deep networks to extract meaningful representation. The data fidelity term promotes data consistency using the hologram formation model.

Overall, our main contributions are as follows:

• We propose a completely new semi-trained approach (HDPhysNet) that combines learned knowledge and physics consistency using the PnP model to solve the challenges of limited resolution and twin image-free phase reconstruction in single-shot LDIHM.
• We use HDGAN and train it on synthetic low-resolution holograms to extract phase information and upsample the reconstruction by 2×.
• We use ADMM to integrate the benefits of HDGAN into the physics-aware untrained deep network (UNet) to refine and improve the results. Furthermore, it maintains more interpretability on unknown samples than the supervised deep methods alone.
• We demonstrate the improved performance of HDPhysNet against the perturbation of imaging parameters such as propagation distance, imaging sample type, and wavelength. We comparatively evaluate the accuracy, efficiency, and generalization capabilities of HDPhysNet over conventional methods, trained DL methods, and physics-aware untrained deep networks in hologram reconstruction.

## 2 Principles of LDIHM Imaging

**Fig. 2:**
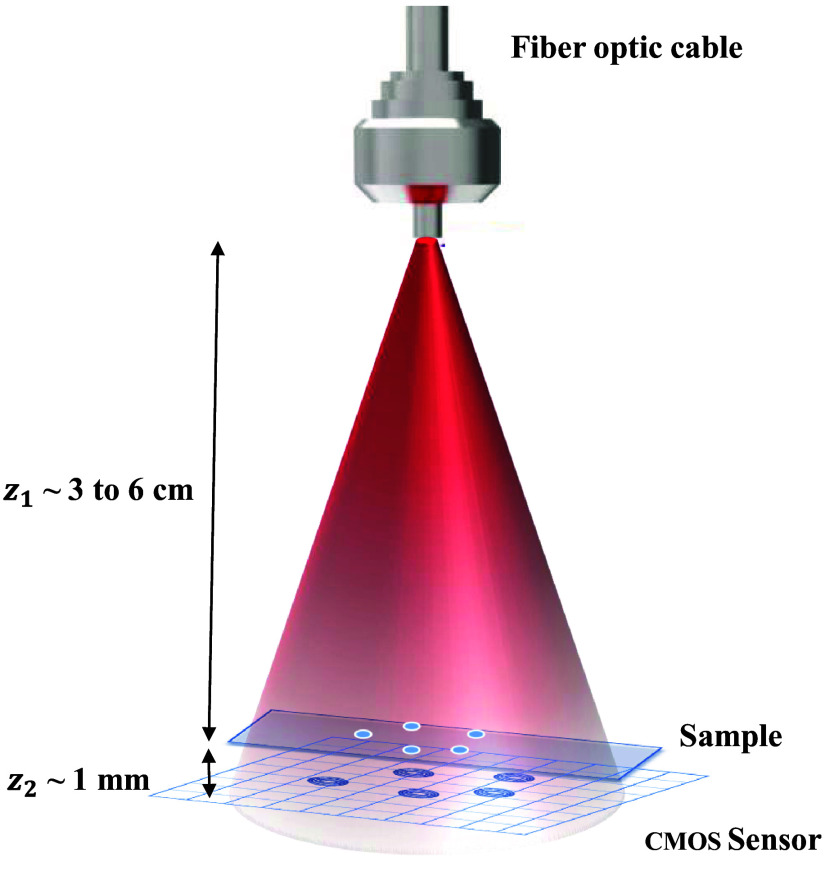
Schematic of the experimental setup of LDIHM.

The sample under imaging is illuminated by a partially coherent light emitting diode (LED) light source of wavelength 627 nm, butt-coupled to an optical fiber (Model M15L01, Thorlabs, Newton, New Jersey, United States) with a coupling efficiency of 16.66%.^8^ The wave diffracted by the weakly absorbing object is represented as the object wave $\left(u_{o}\right)$, and the un-scattered wave acts as the reference wave $\left(u_{r}\right)$ in the object plane. A large source-to-sample distance ($z_{1}∼3-6\;\mathrm{cm}$) and a small sample-to-detector distance (usually in the range of mm) result in an FOV of $∼29{\;\mathrm{mm}}^{2}$. The schematic of the setup is shown in Fig. 2.

The diffracted wavefront in the far field (sensor plane) is given by the Huygens–Fresnel integral as

$$U_{o}(x,y)=\iint u_{o}(\xi ,\eta )\exp(ikz)h(x-\xi ,y-\eta )d\xi \;d\eta ,$$ (1)

where h(.) is the function of free-space propagation of the diffracted object wave $u_{o}(\xi ,\eta )$ along the optical axis with the wave number k=2π/λ (with λ being the wavelength of the propagating wave). The propagation path length, z, is calculated using an autofocusing algorithm.^40^ The reference wave is the recording without a sample, and it is used to normalize the hologram. The interference of the object and reference wave is captured by the complementary metal oxide semiconductor (CMOS) camera with a pixel size of 1.67  μm and a resolution of 3840×2784  pixels. The distribution of these two waves at the detector in the plane (x,y) is described as $U_{o}$ and $U_{R}$, respectively. The hologram intensity is given as

$$I=|U_{o}+U_{R}{|}^{2}=|U_{o}{|}^{2}+|U_{R}{|}^{2}+U_{o}^{*}U_{R}+U_{o}U_{R}^{*}.\;$$ (2)

After normalization, the effect of $|U_{R}{|}^{2}$ is removed, and the non-linearity due to $|U_{o}{|}^{2}$ is modeled as an error e; then, the mapping between the object field and hologram is represented as

$$I=U_{o}^{*}U_{R}+U_{o}U_{R}^{*}+e=H(u_{o}),$$ (3)

where H(.) represents a mapping function that relates the object field $u_{o}$ to the hologram I.

The second step of holography is reconstructing the original object from the captured interference pattern, which is done by backpropagation of the hologram to the object plane. The propagation to the object plane is explained by the Fresnel–Kirchhoff integral,^41^ described as

$$u_{o}(\xi ,\eta )=\frac{i}{\lambda }\iint U_{R}^{*}(x,y)I(x,y)\frac{\exp(-ik\rho )}{\rho }dx\;dy,$$ (4)

where $\rho =\sqrt{(x-\xi {)}^{2}+(y-\eta {)}^{2}+z^{2}}$ is the distance between the hologram plane and the object plane and z is the perpendicular distance between the hologram and the reconstruction plane. The incident plane wave at the object location is obtained as “1” by selecting the optical axis along the propagation of a digital hologram. Then, Eq. (4) is rewritten as

$$u_{o}(\xi ,\eta )=\frac{i}{\lambda }\iint I(x,y)\frac{\exp(-ik\rho )}{\rho }dx\;dy.$$ (5)

The reconstructed complex object u^o(ξ,η) at a distance z from the image sensor using angular spectrum wave propagation^41^ is represented as

u^obj(ξ,η)=F−1[F(I).exp(−2πizλ)1−(λfx)2−(λfy)2], (6)

where F and $F^{-1}$ are Fourier transform and inverse Fourier transform, respectively, and $f_{x}$ and $f_{y}$ are the spatial frequency coordinates. Amplitude and phase are extracted from the reconstructed complex object.

The resolution of the LDIHM depends on several factors: the NA, sample-to-source distance, the degree of the optical coherence (temporal and spatial), and the pixel size of the camera.^7^^,^^42^ The temporal coherence length $\Delta L_{c}$ of the source is calculated as $\Delta L_{c}=(\frac{2\;\ln\;2}{\pi }).\frac{\lambda^{2}}{n.\Delta \lambda }$, where n is the refractive index of the medium, λ is the wavelength, and Δ is the spectral width of the illuminating source. The spatial coherence diameter at the sample plane is proportional to $D_{\mathrm{coh}}=\lambda .z_{1}/D$, where D is the aperture size at the illumination plane.

The resolution imposed by the temporal coherence of illumination is given as $\theta_{\max}\leq \arccos(\frac{z_{2}}{z_{2}+\Delta L_{c}})$. The resolution imposed by spatial coherence is given by $\theta_{\max}\leq \arctan(\frac{0.61\lambda z_{1/D}}{z_{2}})$. Based on these calculations, for a $z_{2}∼1\;\mathrm{mm}$ and spectral bandwidth Δλ∼20  nm, the resolution (Δx) is close to the pixel size ∼1.67  μm.

## 3 Methods

**Fig. 3:**
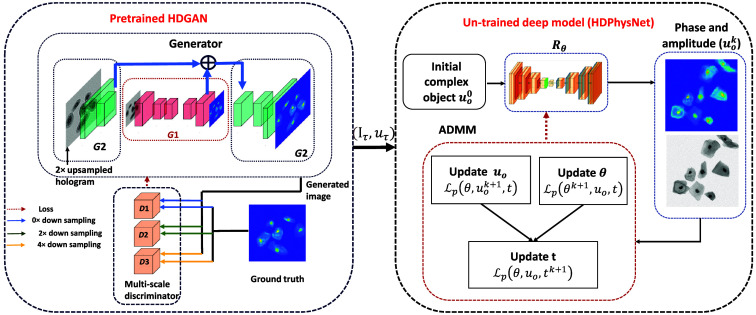
Schematic of the proposed method: a semi-trained deep framework for high-resolution hologram reconstruction.

The low-resolution hologram is recorded under the proposed LDIHM setup, and the high-resolution complex object information (amplitude and phase) is obtained using the proposed semi-trained framework. The proposed semi-trained framework consists of two components: the pre-trained HDGAN and the HDPhysNet. The HDGAN is pre-trained on the simulated dataset to reconstruct a high-definition phase image from a low-resolution diffraction pattern. The HDGAN^39^ can increase the image resolution by 2× or 4× without losing fine details. The HDPhysNet is an extension of our earlier work DIP-RED^31^ in which different denoisers are plug-and-play into physics-aware untrained networks. Here, instead of denoisers, the high-resolution phase generated by the HDGAN is plugged as the learned prior knowledge to further fine-tune the reconstruction through the physics-aware untrained model. This approach combines the benefits of both pre-training and physics-aware task-specific training to enhance the model’s performance. Each component of Fig. 3 is described in Secs. 3.2 and 3.3.

### 3.2 High-Definition Generative Adversarial Networks

The deep network is trained to learn the mapping of the input hologram (I) to the reconstructed phase (Ø) of the object ($u_{o}$). Traditional GAN models often struggle to generate high-resolution images due to the limitations of the network architecture and training process.^43^ HDGANs^39^ incorporate various techniques and modifications to enhance the generation of high-resolution images. We adopted HDGAN to generate high-resolution phase images from low-resolution holograms. With the help of a multiscale generator and discriminator, as shown in Fig. 3, the HDGAN can synthesize phase images of size 1024×1024, which are more visually appealing than those generated by earlier GANs.

To generate high-resolution images, the generator (G) is divided into two sub-networks: $G=\{G_{1},G_{2}\}$. We first train a residual network $G_{1}$, which operates at a resolution of 512×512. Then, another residual network $G_{2}$, which is a local enhancer appended to $G_{1}$, and the two networks are trained jointly on high-resolution images.^39^ Specifically, the feature map of the generator, $G_{1}$, is added to the feature map of the local enhancer $\left(G_{2}\right)$, which ensures the propagation of local information to the global generator. To differentiate the high-resolution real and synthesized images, multiscale discriminators $\left(D_{k}\right)$ are proposed to operate at different image scales. In this study, we used three discriminators of the same architecture, i.e., patchGAN and image scales are obtained by down-sampling them by a factor of 2 and 4. In addition to the multiscale discriminator, the feature matching loss is also calculated at multiple layers of the discriminator.

Using a two-level generator effectively aggregates global and local information for image generation. Also, the multi-scale discriminators are trained at three different scales, which encourages the generator to produce finer details with global consistency. Training for the HDGAN requires a longer time, which is however a one-time effort, leading to high-speed reconstruction after training. HDGAN can produce high-resolution phase images $\left(\phi_{\tau }\right)$ from low-resolution holograms, which is highly desirable for lensless holography.

### 3.3 Physics-Aware Semi-trained Deep Framework

Deep image prior (DIP) is the first untrained network implemented; it incorporates a physical model (forward propagation) of the image formation process in the loss function.^44^ Here, the loss is calculated as the difference between the captured hologram and the generated hologram (generated using the forward model). The untrained network uses ADMM^16^ to optimize the network parameters. ADMM is a variable splitting approach that can incorporate various types of regularization or constraints to promote desirable properties in the reconstructed hologram. ADMM allows for distributed optimization of large problems by dividing them into smaller and more manageable chunks. It is the best way to plug the learned prior into a loss function as a regularization. It is worth noting that the hologram used is a high-resolution hologram $\left(I_{\tau }\right)$ that is obtained from HDGAN for further processing. The detailed description of the proposed HDPhysNet, shown in Fig. 3, is as follows.

#### 3.3.1 Initialization

The first input, ($u_{o}^{0}$), is obtained by backpropagating the high-resolution hologram $\left(I_{\tau }\right)$ to the object plane using the propagation kernel. The phase generated by HDGAN, $\phi_{\tau }$, is combined with a constant amplitude of $u_{o}^{0}$ to form a trained complex object, $u_{\tau }$. Other parameters such as network parameters, θ, are set randomly; the Lagrange multipliers vector, t, is set to 0; and k=0.

#### 3.3.2 Loss function

Network $R_{\theta }$ learns to fine-tune the initial reconstruction $\left(u_{o}^{0}\right)$ using physics consistency and the learned prior $\left(u_{\tau }\right)$. The loss function using ADMM for the proposed HDPhysNet is defined as

$$L_{\mathrm{HD}\text{ PhysNet}}(\theta ,u_{o},t)=\underset{u_{o},\theta }{\arg\;\min}\{\frac{1}{2}{\left\|HR_{\theta }(u_{o})-I_{\tau }\right\|}_{2}^{2}+\frac{\alpha }{2}u_{o}^{T}(u_{o}-u_{\tau })+\frac{\beta }{2}{\left\|u_{o}-R_{\theta }(u_{o})-t\right\|}_{2}^{2}\},$$ (7)

where function H(.) maps complex object (u^o) to the hologram $I^{\prime }$. α and β are regularization parameters. In Eq. (7), the first term is the data fidelity term, which enforces physics consistency by estimating the error between the captured hologram and the generated hologram. The second term is regularization in which the output of the HDGAN is used to regulate the reconstructed object. The gradient of regularization is $u_{o}-u_{\tau }$. The last term is proximity regularization, which forces network output Rθ(u^o) to be close to $u_{o}-t$.

#### 3.3.3 Optimization

ADMM is the variable splitting approach that updates the parameters θ, t, and $u_{o}$, sequentially. θ is updated by fixing t, and $u_{o}$ is given as

$$L_{\theta }(\theta^{k+1},u_{o},t)=\underset{u_{o},\theta }{\arg\;\min}\{\frac{1}{2}{\left\|HR_{\theta }(u_{o})-I_{\tau }\right\|}_{2}^{2}+\frac{\beta }{2}{\left\|u_{o}-R_{\theta }(u_{o})-t\right\|}_{2}^{2}\}.$$ (8)

The effect of the second term in Eq. (7) is negligible while updating θ as $u_{o}$ is kept constant. The next input to the network $u_{o}^{k+1}$ is updated using the steepest descent method, which takes the gradient of the second and the third term in Eq. (7) as θ and t are constants.

$$L_{u_{o}}(\theta ,u_{o}^{k+1},t)=u_{o}^{k}-c[\alpha (u_{o}^{k}-u_{\tau })+\beta (u_{o}^{k+1}-R_{\theta }(u_{o}^{k})-t)].$$ (9)

Here, the residual image is calculated to penalize the object estimate based on learned regularization and proximity regularization. c is the step size that ensures the steepest descent. Finally, the Lagrange multiplier vector t is updated by keeping θ and $u_{o}$ constant:

$$L_{t}(\theta ,u_{o},t^{k+1})=t^{k}-(u_{o}^{k+1}+R_{\theta }(u_{o}^{k})).$$ (10)

The use of the variable splitting method allows for dealing with the error term and the regularization separately; hence, Eqs. (8) and (9) can be executed in parallel to reduce the convergence time.

#### 3.3.4 Convergence/stopping criteria

As discussed, ADMM updates three variables, θ, t, and $u_{o}$, sequentially. Hence, we need a stopping criterion to avoid overfitting. We adapted the stopping criteria from Ref. 45, given as $\max\{\epsilon_{1},\epsilon_{2},\epsilon_{3}\}\leq \mathrm{tol}/3$,

where $\epsilon_{1}={\left\|L_{\theta }(\theta^{k+1},u_{o},t)-L_{\theta }(\theta^{k},u_{o},t)\right\|}_{2}/\sqrt{n}$, $\epsilon_{2}={\left\|L_{u_{o}}(\theta ,u_{o}^{k+1},t)-L_{u_{o}}(\theta ,u_{o}^{k},t)\right\|}_{2}/\sqrt{n}$, and $\epsilon_{3}={\left\|L_{t}(\theta ,u_{o},t^{k+1})-L_{t}(\theta ,u_{o},t^{k})\right\|}_{2}/\sqrt{n}$ for $u_{o}\in R^{n}$. The tolerance level is decided based on the phase SNR value of the reconstructed phase image. We observed that $\mathrm{tol}\approx 10^{-3}$ is sufficient for the convergence of HDPhysNet to avoid overfitting of the interference-related noise.

## 4 Materials

### 4.1 Sample Preparation

Biological samples were collected from the hospital using approved standard protocols accepted through the Institutional Internal Review Committee (IITH/IEC/2024/02/12). Cervical cells of healthy patients were extracted and suspended in a Carnoy’s fixative (methanol: glacial acetic acid = 3:1) solution without any staining procedure. The buffer solution was then dropped on the microscopic slide and imaged under LDIHM. Similarly, the blood sample of a healthy patient was collected and centrifuged to separate red blood cells (RBCs) from the blood. RBCs were diluted with distilled water and dropped on the slide for imaging.

### 4.2 Dataset

The cervical cell dataset was obtained from the ISBI 2014 dataset,^46^ which was designed for the purpose of a segmentation challenge contest (segment overlapping cells). The diffraction patterns are generated with different possible propagation distances used during experiments, e.g., z={0.8,0.9,1,1.1,1.2,1.3}  mm and the wavelength of the illuminating source, λ=627  nm. A phase value of 4 radians was added during the simulation process. The generated hologram and corresponding phase image were stored in different folders with the same name. Out of 2048 images, 1800 images were used for training, 148 for validation, and 100 for testing. It is important to note that the imaging parameters (propagation distance and wavelength) for test images are not the same as those used in the training dataset.

### 4.3 Network Architecture

We used two different networks: HDGAN for the trained model and UNet for the untrained model. HDGAN has a generator and discriminator networks. Note that we have two generators $G=\{G_{1},G_{2}\}$. This network contains two stride-2 convolutions, several residual blocks, and two 1/2-strided convolutions.^39^ The multiscale discriminator has three discriminators working on different image scales. Each discriminator has the same architecture. It has two convolutional layers that work on patches; hence it is called patchGAN. The UNet architecture is adopted from our previous work.^31^

We implemented our untrained model with UNet architecture using the PyTorch framework on a GPU workstation with two NVIDIA GeForce GTX 1080ti 11 GB graphics cards. The HDGAN took 1 day and 18 h for training on 1750 images of 512×512  pixels with 200 epochs. The inference time of trained HDGAN for a hologram of 512×512  pixels is ∼2  s. The reconstruction of a complex object by UNet takes ∼3  min with 700 epochs on simulation data and ∼5.3  min with 1000 epochs on experimental holograms. The dataset preparation and data plotting are done in MATLAB R2022a.

## 5 Result and Discussion

**Fig. 4:**
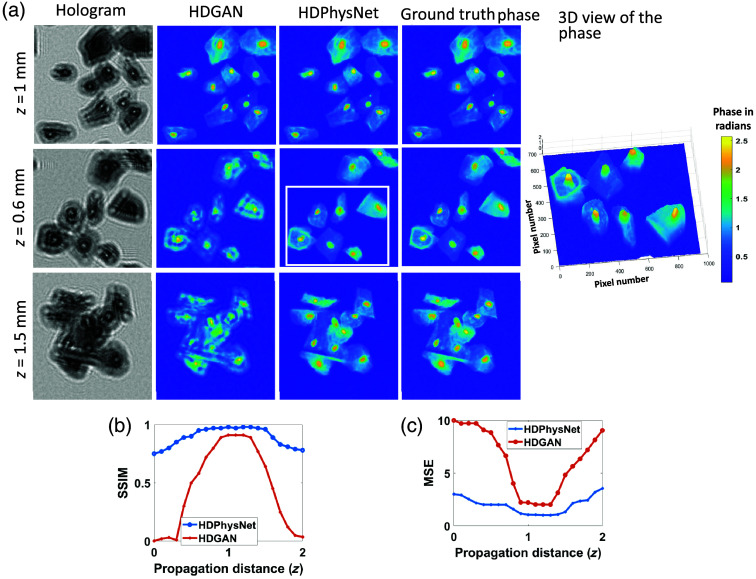
Simulation results: (a) phase reconstruction by HDGAN and HDPhysNet by varying simulation parameters such as propagation distance (Δz in mm). (b) SSIM plot (c) MSE plot.

Generative networks can learn to generate high-definition hologram reconstructions by learning intricate details, complex patterns, and textures and extracting meaningful representations from the data. We used HDGAN^39^ to generate a high-resolution phase from the low-resolution input hologram. The network is trained on the computer-generated holograms. The trained networks are not aware of the imaging physics, which results in low interpretability of unknown samples. The HDPhysNet combined the capabilities of the trained network and the physics of holography to improve the performance on unknown samples and change in imaging parameters (propagation distance and wavelength). To demonstrate this claim, the HDGAN is trained on the cervical cell dataset, which is simulated on experimental parameters, as given in Sec. 4.2. The inference is performed on the samples by varying the propagation distance and the illumination wavelength. It can be observed from Fig. 4(a) that the performance of HDGAN degrades for propagation distances beyond the range given during the training, but HDPhysNet shows consistent performance against the change of these parameters. The first row of Fig. 4(a) indicates that the negligible refinement by HDPhysNet is required on the results of HDGAN as the propagation distance is seen during training. The second and third rows show that the HDGAN could retrieve the structural features from the diffraction pattern with an unseen propagation distance. This initial feature extraction is beneficial for further reconstruction process but needs refinement. HDPhysNet uses this phase as *a priori* knowledge and plugs it with the loss function of the physics-aware network to further fine-tune the reconstruction.

The average structural similarity index metric (SSIM) and mean squared error (MSE) are calculated on 100 test images by HDGAN (trained)^39^ and HDPhysNet (semi-trained). Phase images with SSIM∼1 and very low MSE indicate the highest structural similarity with the ground truth phase image with less error. Figures 4(b) and 4(c) show the SSIM and MSE of HDGAN and HDPhysNet with respect to the propagation distance, respectively. Here, for simplicity, we plotted the SSIM and MSE of only 21 images with varying propagation distances. These graphs indicate that the width of the peak performance is increased by the HDPhysNet over HDGAN because of the physics-based constraints that guide the learning process, making it capable of reconstructing defocused holograms. The results show that the SSIM is increased by 0.07 over HDGAN and 0.04 over DIP-RED. The MSE is decreased by 1.7 over HDGAN and 1.2 over DIP-RED. The proposed HDPhysNet leverages the general representations learned during pre-training using a limited dataset while fine-tuning the reconstruction using physical consistency.

**Fig. 5:**
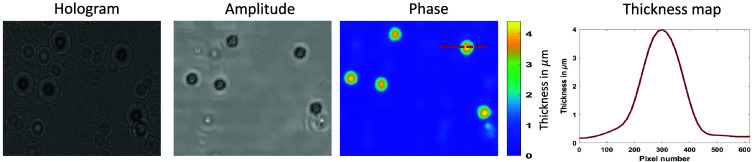
Phase characterization and thickness profile of microbeads (4  μm).

It is worth noting that the GAN considers the phase image to be an intensity (real) image; hence, scaling (phase to intensity) and re-scaling (intensity to phase) are required at the beginning and end of HDGAN. Hence, we used another UNet architecture to handle complex data (amplitude and phase) and used the generated phase as the regularization for better feature extraction and early convergence. The phase characterization of HDPhysNet is performed using the polystyrene microbeads of size 4  μm with a refractive index of 1.68 and a refractive index of the medium (distilled water) of 1.33. The phase is directly proportional to the thickness (h) of the sample and is calculated as $h=\frac{p*\lambda }{2\pi (n_{1}-n_{2})}$ where p is the estimated phase, λ is the wavelength of the illuminating source, and $n_{1}$ and $n_{2}$ are the refractive indices of the sample and surrounding media, respectively. The scaling factor is identified by mapping the phase of the microbeads to the thickness, as shown in Fig. 5. The average thickness calculated on beads is 4±0.05  μm with an error of 0.05  μm.

**Fig. 6:**
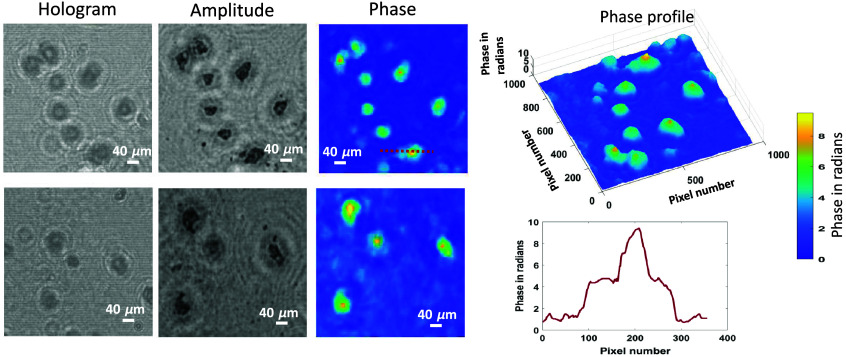
Experimental cervical cells reconstruction; amplitude and phase reconstruction by the proposed method.

The performance of HDPhysNet is observed on experimental hologram images under the proposed LDIHM. The large FOV (3840×2784) experimental holograms of the pap smear sample are captured under our LDIHM setup. Then, the region of interest is cropped and resized to the size 512×512 from the large FOV hologram. The 2× up-sampled phase and hologram are obtained from pre-trained HDGAN. Then, the untrained model uses this information to reconstruct the complex object. The reconstructed phase and amplitude of size 1024×1024 by HDPhysNet are shown in Fig. 6. A 3D view of the phase and the phase profile of the cell are shown in the last column of Fig. 6.

**Fig. 7:**
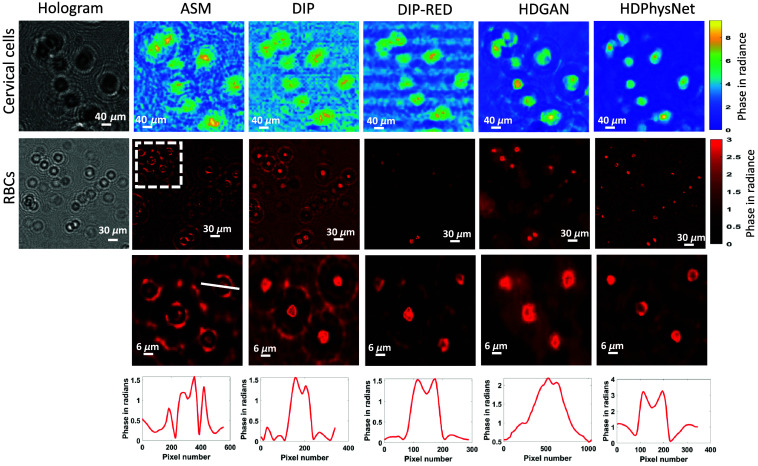
Phase reconstruction on experimental holograms of biological samples: a comparative study of phase recovery by ASM,^41^ DIP,^25^ DIP-RED,^31^ HDGAN,^39^ and the proposed HDPhysNet. Color bars on the right represent phase in radians. We changed the color map of the RBC phase for better visibility.

The reconstructed phase is compared with different existing single-shot hologram reconstruction methods proposed in the literature. The angular spectrum wave propagation (ASM) is the basis for numerical simulation and reconstruction of the hologram, and it is used in many hologram reconstruction methods.^41^ We compared the reconstruction by HDPhysNet with trained (HDGAN) and untrained methods (DIP) separately. We also compared results with our previous work DIP-RED^31^ in which DIP is powered with regularization by denoising to remove interference-related noise and twin images. The denoiser used here is a total variation denoiser. It can be observed from Fig. 7 that DIP-RED has better performance compared with ASM, DIP, and HDGAN. The interpolation used in ASM, DIP, and DIP-RED to increase the visibility introduces some unknown artifacts. This problem is overcome using HDGAN in the proposed method, which upsamples phase and hologram by 2× without losing finer details. Here, the 2× upsampled hologram by the HDGAN is used for reconstruction by each method.

The interpretability of the HDPhysNet on unknown samples is checked on RBCs, which were not part of the training process. It can be observed from Fig. 7 that the results of the HDGAN are biased for RBCs due to the limited interpretability of HDGAN on unknown samples. A cytoplasm-like boundary is generated around the RBCs, which is refined by the HDPhysNet. However, the phase recovered by HDGAN is used in the loss function (of HDPhysNet) to obtain the next input to the network in each iteration. Hence, the phase recovered by HDGAN influences the final reconstruction. This can be improved by adding more data into the training dataset with different imaging samples and varying experimental parameters. The phase SNR (p-SNR) computes the ratio of phase values (P) of the signal and noise, in decibels, as $p-\mathrm{SNR}=10\;\log_{10}[\frac{\max(P)-\mu }{\sigma }]$ where μ is the mean and σ is the covariance. The comparative analysis of the average phase-SNR by each phase recovery method is given in Table 1. It is observed that the P-SNR is increased by 8.2 dB over HDGAN and 9.8 dB over DIP-RED on the experimental data.

**Table 1:** Phase SNR (p-SNR) calculated on reconstructed phase images.

Sample	$z_{2}$ (mm)	ASM	DIP	DIP-RED	HDGAN	HDPhysNet
Simulations	1	43.7	58	58.3	61.2	63.6
Cervical cells	1.3	15.5	24	25.4	32	41.3
RBCs	1.05	12.7	21.8	28.3	25	38

It is worth noting that the sample-to-detector distance for each experiment was ∼1  mm. While carrying out reconstructions, our reconstruction algorithm employs an autofocus algorithm^40^ to calculate the exact propagation distance for the best image quality. Hence, within this range of propagation distance, i.e., 1 to 1.5 mm, the performance of our algorithm is very robust. Simulation data showing the performance of the algorithm versus propagation distance (0 mm to 2 mm) are illustrated in Fig. 4. The experimental results showing reconstructions of two different cell types (RBC and cervical cells) for varying propagation distances are illustrated in Fig. 7. Also, to prove the improved performance of the proposed HDPhysNet against the wavelength of the illuminating source, the detailed analysis is demonstrated in the Supplementary Material. However, to prove the robustness of the HDPhysNet against unknown perturbations in imaging parameters and imaging samples, analysis is needed on a large number of samples.

We used two different deep networks in the hologram reconstruction process. The existing semi-trained approaches use a single network that is trained on a limited dataset along with physics consistency.^33^^,^^38^ The ability of HDGAN to generate high-definition phase images with a limited dataset is appealing over other networks. However, HDGAN has limitations in handling complex data types (amplitude and phase). To address this limitation, we implemented a physics-aware untrained UNet to reconstruct complex objects and used the phase generated by HDGAN as a learned prior. The use of ADMM for untrained network optimization allowed us to use the phase generated by HDGAN efficiently in the loss function without explicit differentiation of regularization. HDPhysNet combines the strengths of a trained deep model, such as its ability to learn complex patterns, with the physics-aware untrained network to give better results, which is difficult to achieve by either DL models (trained or untrained) alone. This can help overcome challenges such as limited hologram training data and the complexity of the holographic reconstruction process.

LDIHM in combination with the proposed HDPhysNet can overcome the limits of the current cytology procedure at point-of-care with the following advantages:

• Label-free, single-shot holography with a large FOV results in the scanning of complex data in a short period.
• HDPhysNet is not sample-specific and, hence, can be successfully generalized to reconstruct unknown biological samples.
• HDPhysNet is stable against the perturbation of the imaging parameters and hence can overcome the stringent requirement of environmental stability.
• Portable and technology-driven microscopy, fast scanning, and early disease diagnosis will revolutionize healthcare in rural areas.

## 6 Conclusion

DL-based hologram reconstruction models are object-specific without any guarantee of performance on unknown practical data. The proposed HDPhysNet for hologram reconstruction is a hybrid approach that combines both DL techniques and the physics of holography. The HDGAN used for phase retrieval increases the resolution without losing finer details and inherently denoises the reconstruction for interference-related noise. The use of ADMM allows us to integrate the benefits of HDGAN into the physics-aware untrained deep network (UNet) to refine and improve the results. The simulation results show that the SSIM of the HDPhysNet is increased by 0.07 over HDGAN (trained) and 0.04 over the untrained method (DIP-RED). The phase-SNR is increased by 8.2 dB over HDGAN and 9.8 dB over DIP-RED on the experimental data. The performance of the HDPhysNet is independent of the experimental parameters and imaging sample, and therefore, it is best suited for point-of-care cytology applications. The inference time of the HDPhysNet can be reduced drastically by adding a small amount of experimental ground-truth data while training. We strongly believe that the HDPhysNet can be easily adapted to other phase imaging modalities by replacing the mapping function (i.e., the forward imaging model).

## Supplementary Material



## Data Availability

Code and data used in this paper can be obtained from the corresponding author upon reasonable request.
