# Supplementary material for: High-resolution lensless holographic microscopy using a physics-aware deep network
Source: J Biomed Opt. 2024 Oct 8;29(10):106502. doi: 10.1117/1.JBO.29.10.106502 (PMC11460617; doi:10.1117/1.JBO.29.10.106502)
Supplement: Supplementary file 1 [file JBO_029_106502_SD001.docx]

**Supplementary material**

The improved performance of the proposed HDPhysNet against the propagation distance and hologram size is demonstrated in the manuscript. To show the robustness against the wavelength of the illuminating source, we have added discussion and results on simulated and experimental cervical cells.

**Simulation Results**

We have simulated holograms illuminated with red (627nm), green (540 nm), and blue (470 nm) light. During the training holograms are simulated with the illumination by only red light. However, the inference is done on the samples illuminated by red, green, and blue light. The reconstruction by HDGAN shows superior performance for red light (known during training) compared to other wavelengths. However, HDPhysNet overcomes this problem as the wavelength is given in the forward model. Table S1 shows the quantitative analysis of HDGAN and HDPhysNet on simulation data by illuminating with different wavelengths.


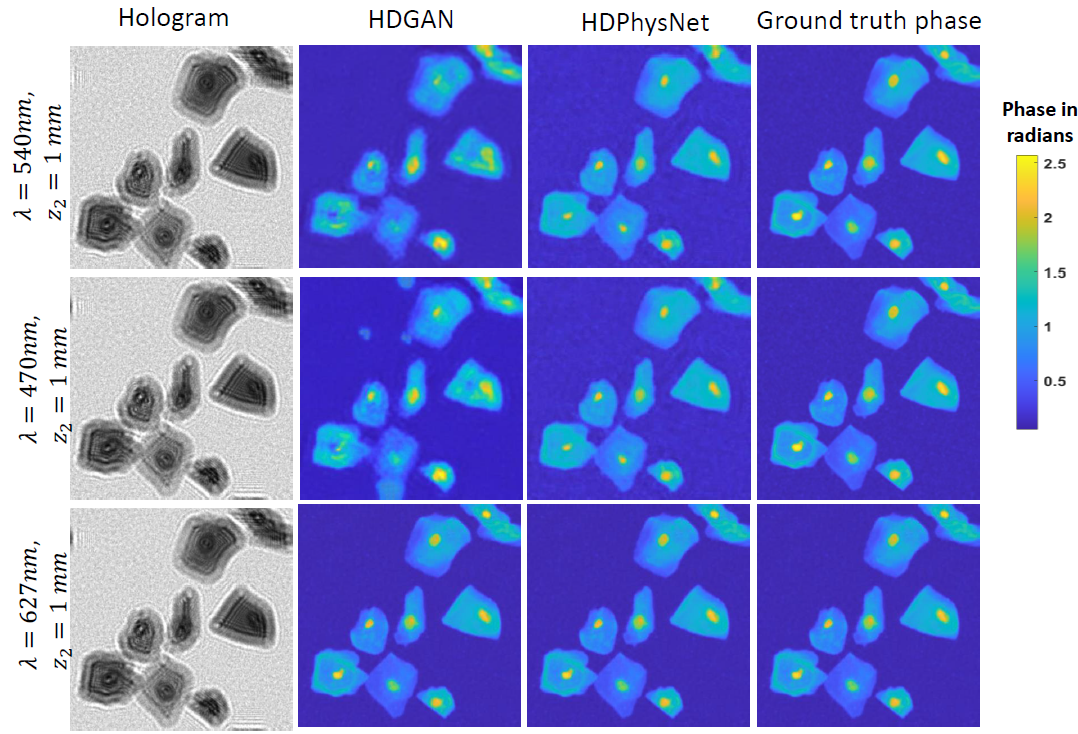


**Fig. S1: Simulation results: (a) Phase reconstruction by HDGAN and HDPhysNet by varying wavelengths of the illuminating light source.**

**Table S1: SSIM and MSE obtained by HDGAN and HDPhysNet for the simulation shown in Fig. S1.**

| **Wavelength (nm)** | **HDGAN** | | **HDPhysNet** | |
| --- | --- | --- | --- | --- |
|  | **SSIM** | **MSE** | **SSIM** | **MSE** |
| 627 | 0.95 | 2.02 | 0.97 | 1.92 |
| 540 | 0.72 | 4.02 | 0.81 | 2.9 |
| 470 | 0.75 | 3.9 | 0.8 | 2.9 |

**Experimental Results**

Similar experiments have been performed on cervical samples imaged under the proposed LDIHM with different wavelengths. Propagation distance $z_{2}$obtained after the autofocus algorithm [53] is 1.3mm. HDGAN shows different reconstructions for each wavelength as the experimental samples are unknown to the HDGAN. However, the HDPhysNet improved the initial reconstruction obtained from the HDGAN in terms of the Phase SNR values as shown in Table S2. Also, the phase profile of the individual cells by HDPhysNet for all wavelengths shows better features than HDGAN. However, to prove the robustness of the HDPhysNet against unknown perturbations in imaging parameters and imaging samples more analysis is needed on a large number of samples.


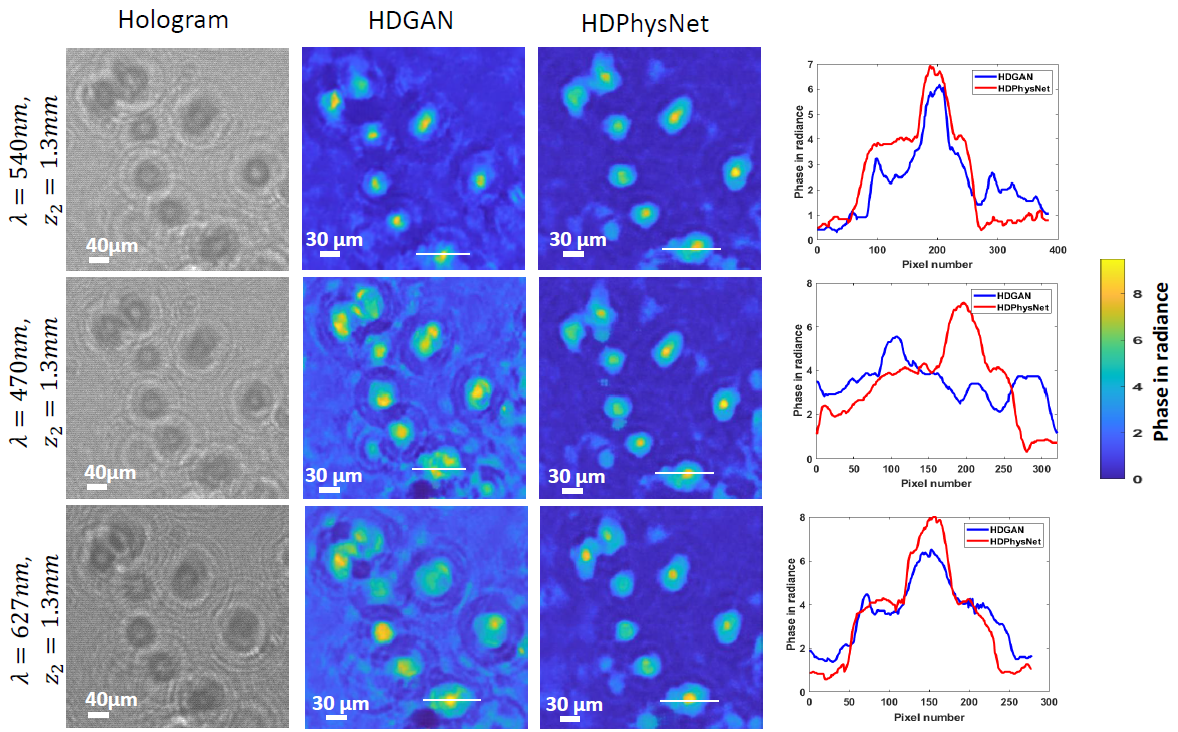


**Fig. S2: Experimental results (cervical cells): (a) Phase reconstruction by HDGAN and HDPhysNet by varying the wavelength of the illuminating light source.**

**Table S2 Phase SNR (p-SNR) calculated on reconstructed phase images**

| **Wavelength (nm)** | **HDGAN** | **HDPhysNet** |
| --- | --- | --- |
| 627 | 32 | 41.3 |
| 540 | 29.5 | 41 |
| 470 | 26 | 39 |
